# 5-Isopropyl-5-methyl-2-sulfanylidene­imidazolidin-4-one

**DOI:** 10.1107/S1600536813013639

**Published:** 2013-05-25

**Authors:** Masaki Ichitani, Soh-ichi Kitoh, Shuhei Fujinami, Mitsuhiro Suda, Mitsunori Honda, Ko-Ki Kunimoto

**Affiliations:** a Division of Material Sciences, Graduate School of Natural Science and Technology, Kanazawa University, Kakuma-machi, Kanazawa 920-1192, Japan

## Abstract

In the title compound, C_7_H_12_N_2_OS, the 2-sulfanylideneimidazolidin-4-one moiety is nearly planar, with a maximum deviation of 0.054 (2) Å. In the crystal, a pair of N—H⋯O hydrogen bonds and a pair of N—H⋯S hydrogen bonds each form a centrosymmetric ring with an *R*_2_^2^(8) graph-set motif. The enanti­omeric *R* and *S* mol­ecules are alternately linked into a tape along [1-10] *via* these pairs of hydrogen bonds.

## Related literature

For applications and the biological activity of 2-sulfanylideneimidazolidin-4-ones, see: Marton *et al.* (1993 ▶). For the crystal structures of related compounds, see: Devillanova *et al.* (1987 ▶); Ogawa *et al.* (2009 ▶); Walker *et al.* (1969 ▶). For a description of the Cambridge Structural Database, see: Allen (2002 ▶). For hydrogen-bond motifs, see: Etter (1990 ▶). For the synthetic procedure, see: Wang *et al.* (2006 ▶).
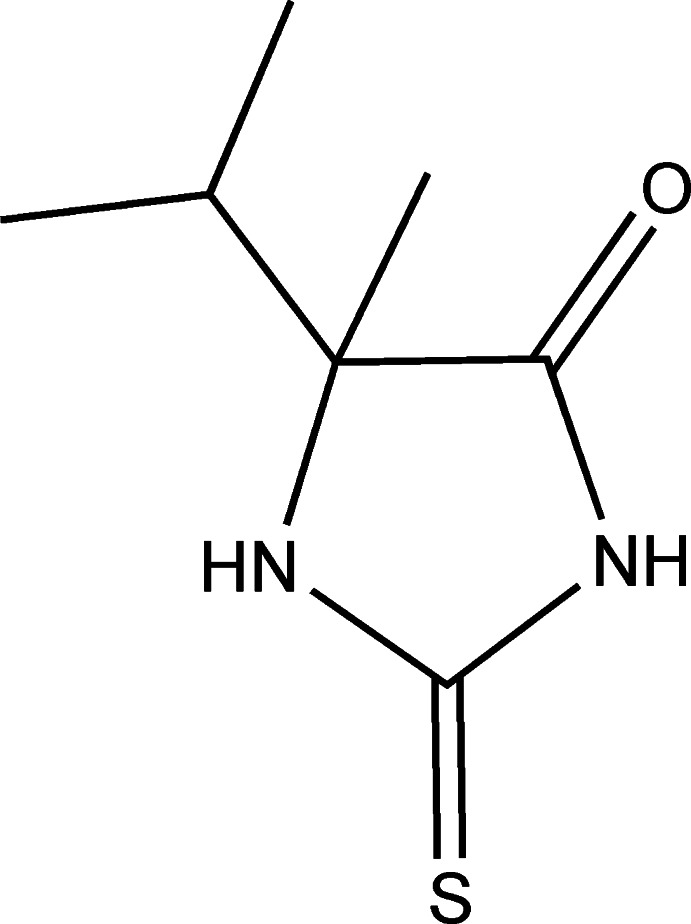


## Experimental

### 

#### Crystal data

C_7_H_12_N_2_OS
*M* *_r_* = 172.26
Monoclinic, 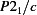

*a* = 5.8317 (5) Å
*b* = 9.2114 (8) Å
*c* = 16.8967 (16) Å
β = 95.855 (3)°
*V* = 902.92 (14) Å^3^
*Z* = 4
Mo *K*α radiation
μ = 0.31 mm^−1^
*T* = 123 K
0.20 × 0.10 × 0.04 mm

#### Data collection

Rigaku/MSC Mercury CCD diffractometer
Absorption correction: multi-scan (*REQAB*; Rigaku, 1998 ▶) *T*_min_ = 0.795, *T*_max_ = 0.988
9484 measured reflections
2051 independent reflections
1660 reflections with *F* ^2^ > 2σ(*F* ^2^)
*R*_int_ = 0.038

#### Refinement

*R*[*F* ^2^ > 2σ(*F* ^2^)] = 0.050
*wR*(*F* ^2^) = 0.126
*S* = 1.14
2051 reflections
111 parameters
H atoms treated by a mixture of independent and constrained refinement
Δρ_max_ = 0.63 e Å^−3^
Δρ_min_ = −0.31 e Å^−3^

### 

Data collection: *CrystalClear* (Rigaku, 2006 ▶); cell refinement: *CrystalClear*; data reduction: *CrystalClear*; program(s) used to solve structure: *SIR2008* in *Il Milione* (Burla *et al.*, 2007 ▶); program(s) used to refine structure: *SHELXL97* (Sheldrick, 2008 ▶); molecular graphics: *ORTEP-3 for Windows* (Farrugia, 2012 ▶) and *Mercury* (Macrae *et al.*, 2006 ▶); software used to prepare material for publication: *CrystalStructure* (Rigaku, 2010 ▶).

## Supplementary Material

Crystal structure: contains datablock(s) global, I. DOI: 10.1107/S1600536813013639/is5273sup1.cif

Structure factors: contains datablock(s) I. DOI: 10.1107/S1600536813013639/is5273Isup2.hkl

Supplementary material file. DOI: 10.1107/S1600536813013639/is5273Isup3.cml

Additional supplementary materials:  crystallographic information; 3D view; checkCIF report

## Figures and Tables

**Table 1:** Hydrogen-bond geometry (Å, °)

*D*—H⋯*A*	*D*—H	H⋯*A*	*D*⋯*A*	*D*—H⋯*A*
N1—H1⋯S1^i^	0.78 (3)	2.63 (3)	3.383 (2)	162 (3)
N2—H2⋯O1^ii^	0.91 (4)	1.93 (4)	2.820 (3)	166 (3)

Symmetry codes: (i) 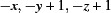; (ii) 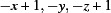.

**Fig. 1.**
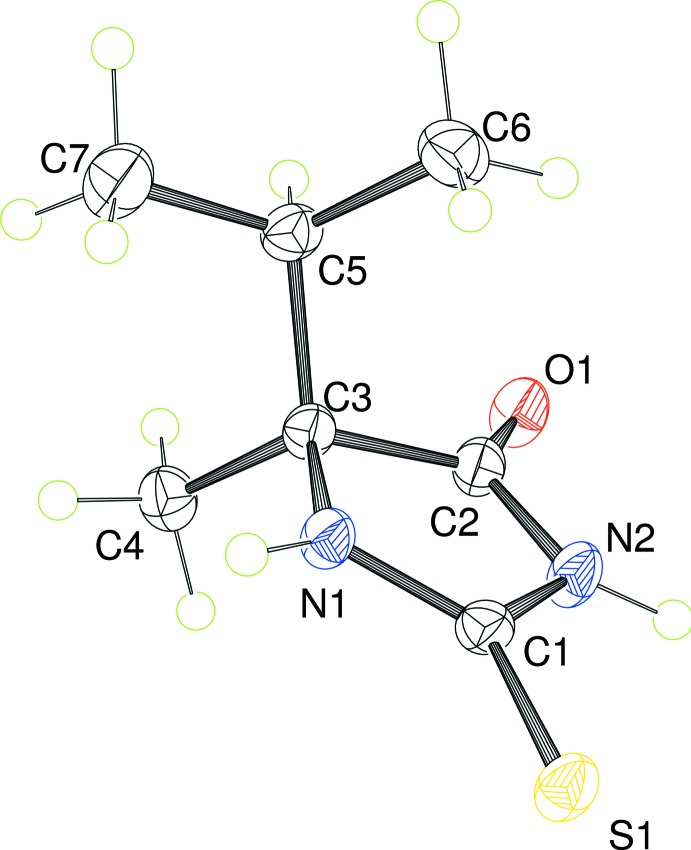
The molecular structure of the title compound with the atom-numbering scheme. Displacement ellipsoids are drawn at the 50% probability level.

**Fig. 2.**
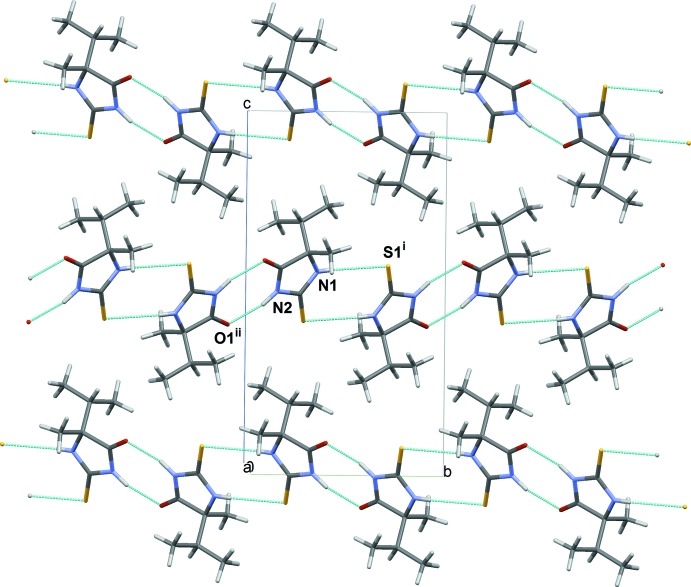
A partial packing diagram of the title compound, viewed down the a axis. Hydrogen bonds are shown as dashed cyan lines (see Table 1 for details).

## supplementary crystallographic information

### Comment

2-Sulfanylideneimidazolidin-4-one (2-thiohydantoin) derivatives are useful synthetic intermediates with a wide range of applications, such as therapeutics, fungicides and herbicides (Marton *et al.*, 1993). Furthermore, 2-sulfanylideneimidazolidin-4-ones have an interesting structural feature. These compounds commonly carry a thioamide and an amide group in a molecule, which provide equal numbers of the proton donor (D) and the acceptor (A) in a D–A–D–A sequence. Because of this unique structural feature, 2-sulfanylideneimidazolidin-4-ones are expected to form intricate hydrogen bonding networks in crystals. We have been studying the polymorphism and molecular conformations of 2-sulfanylideneimidazolidin-4-one (Ogawa *et al.*, 2009) and their derivatives. In this paper, we report on the crystal structure of the title compound, C_7_H_12_N_2_OS.

In the title molecule (Fig. 1), the 2-sulfanylideneimidazolidin-4-one moiety (S1/O1/N1/N2/C1–C3) is nearly planar, with a maximum deviation of 0.054 (2) Å for atom N2. The N1—C1 distance [1.328 (3) Å] is shorter than the N2—C1 distance [1.389 (3) Å], and the S1—C1—N1 angle [128.62 (17)°] is greater than the S1—C1—N2 angle [124.27 (16)°]. These structural features are similar to those observed in 2-thiohydantoin (Devillanova *et al.*, 1987; Ogawa *et al.*, 2009; Walker *et al.*, 1969) and other 2-thiohydantoin derivatives reported in the Cambridge Structural Database (Version 5.34; Allen, 2002) with both unsubstituted NH groups and *sp*^3^-hybridization at C3.

In the crystal structure (Fig. 2), the enantiomeric *R*- and *S*-molecules are connected *via* intermoleculer N1—H1···S1 hydrogen bonds of the neighboring thioamide moieties to form centrosymmetric *R*^2^_2_(8) rings (Etter *et al.*, 1990) (Table 1). Furthermore, the other centrosymmetric *R*^2^_2_(8) rings are formed *via* intermolecular N2—H2···O1 hydrogen bonds of the neighboring amide moieties (Table 1). These two different rings are linked alternately into infinite one-dimensional tapes.

### Experimental

The title compound was synthesized by slight modification of a reported method (Wang *et al.*, 2006). A mixture of α-methyl-*DL*-valine (0.20 g, 1.53 mmol) and thiourea (0.35 g, 4.57 mmol) were allowed to react directly in the absence of any solvent at 180 °C for 5 h. The crude products were further purified by flash column chromatography using hexane and ethyl acetate as eluents (yield: 60%). Colorless crystals suitable for X-ray diffraction analysis were grown by slow evaporation from an aqueous solution.

### Refinement

H atoms bonded to N atoms were located in a difference map and refined freely [N1—H1 = 0.78 (3); N2—H2 = 0.91 (4)]. The remaining H atoms were positioned geometrically (C—H = 0.98 or 1.00 Å) and refined using a riding model, with *U*_iso_(H) = 1.2*U*_eq_(C). A rotating group model was applied to the methyl groups.

### Crystal data

**Table:**

C_7_H_12_N_2_OS	*F*(000) = 368
*M**_r_* = 172.26	*D*_x_ = 1.267 Mg m^−^^3^
Monoclinic, *P*2_1_/*c*	Mo *K*α radiation, λ = 0.71070 Å
Hall symbol: -P 2ybc	Cell parameters from 2692 reflections
*a* = 5.8317 (5) Å	θ = 3.5–27.5°
*b* = 9.2114 (8) Å	µ = 0.31 mm^−^^1^
*c* = 16.8967 (16) Å	*T* = 123 K
β = 95.855 (3)°	Plate, colorless
*V* = 902.92 (14) Å^3^	0.20 × 0.10 × 0.04 mm
*Z* = 4	

### Data collection

**Table:**

Rigaku/MSC Mercury CCD diffractometer	1660 reflections with *F*^2^ > 2σ(*F*^2^)
Detector resolution: 7.314 pixels mm^-1^	*R*_int_ = 0.038
ω scans	θ_max_ = 27.5°, θ_min_ = 3.3°
Absorption correction: multi-scan (*REQAB*; Rigaku, 1998)	*h* = −7→7
*T*_min_ = 0.795, *T*_max_ = 0.988	*k* = −11→10
9484 measured reflections	*l* = −21→21
2051 independent reflections	

### Refinement

**Table:**

Refinement on *F*^2^	Secondary atom site location: difference Fourier map
*R*[*F*^2^ > 2σ(*F*^2^)] = 0.050	Hydrogen site location: inferred from neighbouring sites
*wR*(*F*^2^) = 0.126	H atoms treated by a mixture of independent and constrained refinement
*S* = 1.14	*w* = 1/[σ^2^(*F*_o_^2^) + (0.0462*P*)^2^ + 1.0263*P*] where *P* = (*F*_o_^2^ + 2*F*_c_^2^)/3
2051 reflections	(Δ/σ)_max_ < 0.001
111 parameters	Δρ_max_ = 0.63 e Å^−^^3^
0 restraints	Δρ_min_ = −0.31 e Å^−^^3^
Primary atom site location: structure-invariant direct methods	

### Special details

**Table:**

Experimental. m.p. 145 °C; ^1^H NMR (500 MHz, CDCl_3_): *δ* 9.11 (br s, 1H), 8.09 (br s, 1H), 2.07 (sep, 1H, *J* = 6.9 Hz), 1.46 (s, 3H), 1.03 (d, 3H, *J* = 6.9 Hz), 0.95 (d, 3H, *J* = 6.9 Hz); ^13^C NMR (125 MHz, CDCl_3_): *δ* 181.13, 177.71, 70.15, 34.82, 20.97, 16.86, 16.42; IR (KBr, cm^-1^): 3182 (*ν*(N—H)), 1743 (*ν*(C═O)), 1532 (*ν*(C—N)+*δ*(N—H)).
Geometry. All e.s.d.'s (except the e.s.d. in the dihedral angle between two l.s. planes) are estimated using the full covariance matrix. The cell e.s.d.'s are taken into account individually in the estimation of e.s.d.'s in distances, angles and torsion angles; correlations between e.s.d.'s in cell parameters are only used when they are defined by crystal symmetry. An approximate (isotropic) treatment of cell e.s.d.'s is used for estimating e.s.d.'s involving l.s. planes.
Refinement. Refinement was performed using all reflections. The weighted *R*-factor (*wR*) and goodness of fit (*S*) are based on *F*^2^. *R*-factor (gt) are based on *F*. The threshold expression of *F*^2^ > 2.0 σ(*F*^2^) is used only for calculating *R*-factor (gt).

### Fractional atomic coordinates and isotropic or equivalent isotropic displacement parameters (Å^2^)

**Table:**

	*x*	*y*	*z*	*U*_iso_*/*U*_eq_	
S1	−0.05936 (9)	0.28891 (6)	0.42364 (3)	0.02414 (18)	
O1	0.6264 (3)	0.09152 (17)	0.58845 (10)	0.0278 (4)	
N1	0.2019 (4)	0.3572 (2)	0.55956 (10)	0.0204 (4)	
N2	0.3053 (4)	0.1562 (2)	0.50439 (11)	0.0244 (5)	
C1	0.1488 (4)	0.2702 (3)	0.49775 (12)	0.0192 (5)	
C2	0.4673 (4)	0.1723 (3)	0.56850 (13)	0.0219 (5)	
C3	0.4068 (4)	0.3105 (3)	0.61121 (12)	0.0190 (5)	
C4	0.6026 (4)	0.4209 (3)	0.61059 (14)	0.0259 (5)	
C5	0.3484 (4)	0.2730 (3)	0.69649 (12)	0.0225 (5)	
C6	0.1683 (5)	0.1524 (3)	0.69600 (14)	0.0300 (6)	
C7	0.2715 (5)	0.4046 (3)	0.74022 (15)	0.0331 (6)	
H1	0.140 (5)	0.431 (4)	0.5650 (16)	0.026 (7)*	
H2	0.320 (6)	0.086 (4)	0.468 (2)	0.049 (9)*	
H4A	0.7335	0.3896	0.6476	0.0310*	
H4B	0.5490	0.5161	0.6269	0.0310*	
H4C	0.6506	0.4277	0.5568	0.0310*	
H5	0.4929	0.2361	0.7268	0.0270*	
H6A	0.2276	0.0643	0.6726	0.0360*	
H6B	0.0265	0.1833	0.6644	0.0360*	
H6C	0.1355	0.1321	0.7507	0.0360*	
H7A	0.1359	0.4476	0.7100	0.0397*	
H7B	0.3966	0.4761	0.7460	0.0397*	
H7C	0.2323	0.3755	0.7930	0.0397*	

### Atomic displacement parameters (Å^2^)

**Table:**

	*U*^11^	*U*^22^	*U*^33^	*U*^12^	*U*^13^	*U*^23^
S1	0.0281 (3)	0.0216 (3)	0.0209 (3)	0.0062 (3)	−0.0060 (2)	−0.0030 (2)
O1	0.0281 (9)	0.0245 (9)	0.0290 (9)	0.0104 (7)	−0.0057 (7)	−0.0047 (7)
N1	0.0255 (10)	0.0156 (9)	0.0193 (9)	0.0067 (8)	−0.0011 (7)	−0.0018 (7)
N2	0.0315 (11)	0.0185 (9)	0.0219 (10)	0.0086 (8)	−0.0039 (8)	−0.0053 (8)
C1	0.0223 (10)	0.0177 (10)	0.0171 (10)	0.0037 (8)	0.0005 (8)	0.0013 (8)
C2	0.0245 (11)	0.0192 (11)	0.0212 (11)	0.0008 (9)	−0.0016 (8)	−0.0017 (8)
C3	0.0205 (10)	0.0178 (10)	0.0179 (10)	0.0024 (8)	−0.0017 (8)	−0.0007 (8)
C4	0.0254 (11)	0.0231 (11)	0.0286 (12)	−0.0014 (9)	0.0001 (9)	−0.0003 (9)
C5	0.0223 (11)	0.0258 (11)	0.0188 (10)	−0.0012 (9)	−0.0004 (8)	0.0001 (9)
C6	0.0306 (12)	0.0296 (13)	0.0295 (12)	−0.0072 (10)	0.0010 (10)	0.0047 (10)
C7	0.0376 (14)	0.0332 (14)	0.0286 (13)	0.0006 (11)	0.0035 (10)	−0.0060 (10)

### Geometric parameters (Å, º)

**Table:**

S1—C1	1.662 (2)	N2—H2	0.91 (4)
O1—C2	1.210 (3)	C4—H4A	0.980
N1—C1	1.328 (3)	C4—H4B	0.980
N1—C3	1.470 (3)	C4—H4C	0.980
N2—C1	1.389 (3)	C5—H5	1.000
N2—C2	1.371 (3)	C6—H6A	0.980
C2—C3	1.523 (3)	C6—H6B	0.980
C3—C4	1.529 (3)	C6—H6C	0.980
C3—C5	1.553 (3)	C7—H7A	0.980
C5—C6	1.528 (4)	C7—H7B	0.980
C5—C7	1.512 (4)	C7—H7C	0.980
N1—H1	0.78 (3)		
			
C1—N1—C3	113.63 (18)	C3—C4—H4A	109.471
C1—N2—C2	112.12 (18)	C3—C4—H4B	109.469
S1—C1—N1	128.62 (17)	C3—C4—H4C	109.469
S1—C1—N2	124.27 (16)	H4A—C4—H4B	109.470
N1—C1—N2	107.11 (18)	H4A—C4—H4C	109.476
O1—C2—N2	126.9 (2)	H4B—C4—H4C	109.473
O1—C2—C3	126.2 (2)	C3—C5—H5	107.349
N2—C2—C3	106.89 (18)	C6—C5—H5	107.356
N1—C3—C2	100.18 (16)	C7—C5—H5	107.359
N1—C3—C4	111.34 (17)	C5—C6—H6A	109.476
N1—C3—C5	111.94 (17)	C5—C6—H6B	109.471
C2—C3—C4	110.09 (18)	C5—C6—H6C	109.465
C2—C3—C5	109.67 (18)	H6A—C6—H6B	109.480
C4—C3—C5	112.89 (17)	H6A—C6—H6C	109.472
C3—C5—C6	111.88 (17)	H6B—C6—H6C	109.463
C3—C5—C7	112.26 (19)	C5—C7—H7A	109.475
C6—C5—C7	110.4 (2)	C5—C7—H7B	109.480
C1—N1—H1	123.2 (19)	C5—C7—H7C	109.472
C3—N1—H1	122.7 (19)	H7A—C7—H7B	109.474
C1—N2—H2	126 (2)	H7A—C7—H7C	109.465
C2—N2—H2	121 (2)	H7B—C7—H7C	109.461
			
C1—N1—C3—C2	−1.1 (3)	O1—C2—C3—C5	−61.8 (3)
C1—N1—C3—C4	115.27 (19)	N2—C2—C3—N1	−0.6 (2)
C1—N1—C3—C5	−117.31 (18)	N2—C2—C3—C4	−117.96 (18)
C3—N1—C1—S1	−176.53 (17)	N2—C2—C3—C5	117.24 (18)
C3—N1—C1—N2	2.4 (3)	N1—C3—C5—C6	58.7 (3)
C1—N2—C2—O1	−178.9 (2)	N1—C3—C5—C7	−66.0 (2)
C1—N2—C2—C3	2.1 (3)	C2—C3—C5—C6	−51.5 (2)
C2—N2—C1—S1	176.16 (17)	C2—C3—C5—C7	−176.24 (15)
C2—N2—C1—N1	−2.9 (3)	C4—C3—C5—C6	−174.68 (16)
O1—C2—C3—N1	−179.6 (2)	C4—C3—C5—C7	60.6 (3)
O1—C2—C3—C4	63.0 (3)		

### Hydrogen-bond geometry (Å, º)

**Table:**

*D*—H···*A*	*D*—H	H···*A*	*D*···*A*	*D*—H···*A*
N1—H1···S1^i^	0.78 (3)	2.63 (3)	3.383 (2)	162 (3)
N2—H2···O1^ii^	0.91 (4)	1.93 (4)	2.820 (3)	166 (3)

Symmetry codes: (i) −*x*, −*y*+1, −*z*+1; (ii) −*x*+1, −*y*, −*z*+1.

## References

[bb1] Allen, F. H. (2002). *Acta Cryst.* B**58**, 380–388. 10.1107/s0108768102003890 12037359

[bb2] Burla, M. C., Caliandro, R., Camalli, M., Carrozzini, B., Cascarano, G. L., De Caro, L., Giacovazzo, C., Polidori, G., Siliqi, D. & Spagna, R. (2007). *J. Appl. Cryst.* **40**, 609–613.

[bb3] Devillanova, F. A., Isaia, F., Verani, G., Battaglia, L. P. & Corradi, A. B. (1987). *J. Chem. Res.* **6**, 192–193.

[bb4] Etter, M. C. (1990). *Acc. Chem. Res.* **23**, 120–126.

[bb5] Farrugia, L. J. (2012). *J. Appl. Cryst.* **45**, 849–854.

[bb6] Macrae, C. F., Edgington, P. R., McCabe, P., Pidcock, E., Shields, G. P., Taylor, R., Towler, M. & van de Streek, J. (2006). *J. Appl. Cryst.* **39**, 453–457.

[bb7] Marton, J., Enisz, J., Hosztafi, S. & Timar, T. (1993). *J. Agric. Food Chem.* **41**, 148–152.

[bb8] Ogawa, T., Okumura, H., Honda, M., Suda, M., Fujinami, S., Kuwae, A., Hanai, K. & Kunimoto, K.-K. (2009). *Anal. Sci. X-ray Struct. Anal. Online*, **25**, 91–92.

[bb9] Rigaku (1998). *REQAB* Rigaku Corporation, Tokyo, Japan.

[bb10] Rigaku (2006). *CrystalClear* Rigaku Corporation, Tokyo, Japan.

[bb11] Rigaku (2010). *CrystalStructure* Rigaku Corporation, Tokyo, Japan.

[bb12] Sheldrick, G. M. (2008). *Acta Cryst.* A**64**, 112–122. 10.1107/S0108767307043930 18156677

[bb13] Walker, L. A., Folting, K. & Merritt, L. L. (1969). *Acta Cryst.* B**25**, 88–93.

[bb14] Wang, Z. D., Sheikh, S. O. & Zhang, Y. (2006). *Molecules*, **11**, 739–750. 10.3390/11100739 PMC6148508 17971750

